# Ribosome-Induced Cellular Multipotency, an Emerging Avenue in Cell Fate Reversal

**DOI:** 10.3390/cells10092276

**Published:** 2021-09-01

**Authors:** Arif Istiaq, Kunimasa Ohta

**Affiliations:** 1 Department of Stem Cell Biology, Faculty of Arts and Science, Kyushu University, Fukuoka 819-0395, Japan; istiaqarif@gmail.com; 2 Department of Brain Morphogenesis, Institute of Molecular Embryology and Genetics, Kumamoto University, Kumamoto 860-8555, Japan; 3 HIGO Program, Kumamoto University, Kumamoto 860-8555, Japan

**Keywords:** ribosome, induced pluripotency, reprogramming, cancer, cellular stress, cancer therapy

## Abstract

The ribosome, which is present in all three domains of life, plays a well-established, critical role in the translation process by decoding messenger RNA into protein. Ribosomal proteins, in contrast, appear to play non-translational roles in growth, differentiation, and disease. We recently discovered that ribosomes are involved in reverting cellular potency to a multipotent state. Ribosomal incorporation (the uptake of free ribosome by living cells) can direct the fate of both somatic and cancer cells into multipotency, allowing them to switch cell lineage. During this process, both types of cells experienced cell-cycle arrest and cellular stress while remaining multipotent. This review provides a molecular perspective on current insights into ribosome-induced multipotency and sheds light on how a common stress-associated mechanism may be involved. We also discuss the impact of this phenomenon on cancer cell reprogramming and its potential in cancer therapy.

## 1. Introduction

Cellular potency refers to the ability of a cell to differentiate into a different cell type. The greater the potency of a cell, the greater the number of possible fates it can adopt. Mammalian development is strongly linked to cellular potency. Cellular potency generally diminishes during development as cells become more specialized to perform specific functions [1,2]. An exception is stem cells, which have varying potency and self-renewal capacities; stem cells are classified as totipotent, pluripotent, multipotent, oligopotent, or unipotent based on their potency. Totipotent stem cells can differentiate into any type of cell in the body, whereas unipotent stem cells can only differentiate into one type of cell. Pluripotent stem cells, with the exception of placenta cells, can generate all three germ-layer-derived cell types [3]. Multipotent stem cells differentiate in a more restricted manner, usually within the same germ-layer cells [4]. Stem cells can produce cells that terminally differentiate into specific cells in the body [5].

Under normal conditions, differentiated cells retain their identity to facilitate proper function in the tissue. However, in some conditions, cells can change their identity, a phenomenon called cellular plasticity [6]. Cellular plasticity is an important feature that enables cells to maintain tissue homeostasis and repair. Such cellular conversion includes two key identity-switching processes, known as de-differentiation and transdifferentiation. In de-differentiation, a differentiated cell reverts to a less differentiated state with increased potency. In transdifferentiation, a cell converts (directly or through less differentiated intermediates) to another type of cell within the same or different germ layers. De-differentiation and transdifferentiation processes are an aspect of the development. Both mammals and invertebrates are known to undergo these processes in response to physiological stress and regeneration [7,8,9,10].

Cellular conversion can also occur under experimental conditions where cells de-differentiate or transdifferentiate and adopt an altered fate (typically known as cell reprogramming). Several in vitro reprogramming approaches have been established since the discovery of John Gurdon’s somatic cell nuclear transfer technique to reprogram differentiated cells [11]; these include embryonic stem (ES) cell fusion, chemical reprogramming, forced expression of transcription factor-mediated induced pluripotent stem cells (iPSCs), direct reprogramming, and ribosome-induced cell reprogramming [9,12,13,14,15,16]. Notably, the generation of iPSCs from terminally differentiated cells by ectopic expression of four transcription factors (Yamanaka factors), OCT4, SOX2, KLF4, and MYC, revolutionized cell reprogramming research by overcoming the ethical barrier and having far-reaching implications [9,17]. In general, iPSCs are created through retroviral gene delivery of Yamanaka factors into terminally differentiated cells such as fibroblasts. RNA and protein delivery can also result in the formation of iPSCs [18]. Despite extensive research on iPSCs, there are still some gaps in the understanding of how these transcription factors trigger the reprogramming process [19]. Elucidating the fundamental processes of cell reprogramming continues to be a challenge in developmental biology. In this review, we focus on comprehensively describing our recently developed in vitro cell reprogramming method that uses exogenous, free ribosomes. To the best of our knowledge, we are the only group to work on this approach, and no similar studies have been reported. Consequently, our assertion in this review requires further validation, which is ongoing.

Ribosomes are macromolecular complexes made of proteins and RNA that serve as translation machines in the cell. Ribosomal proteins, in addition to translation, also play roles in development, differentiation, and cancer, which are referred to as “extra-ribosomal functions” [20,21]. We recently demonstrated that exogenous ribosomal incorporation (the uptake of free ribosome by living cells) into somatic and cancer cells causes the reversal of cell fate into a multipotent state [16,22,23,24]. The functional role of the ribosome as an in vitro de-differentiating factor is a recent discovery in the field of cell reprogramming; however, the molecular mechanism underlying ribosome-mediated multipotency is unknown. At the molecular level, somatic and cancer cells differ in a variety of ways. Cancer cells are less specialized than somatic cells, with uncontrolled proliferation, mutation, and altered epigenetics [25,26]. However, both somatic and cancer cells undergo cell-cycle arrest, sphere formation, and multipotency when subjected to ribosome incorporation, suggesting that a similar mechanism is at work. Such an effect on the cells has the potential to be therapeutic. Inducing cell-cycle arrest and reprogramming cancer cells to become non-cancerous is a promising cancer therapy goal [27].

In this review, we first discuss the ribosome-induced somatic cell multipotency, coupled cellular stress, and the associated partial mesenchymal to epithelial transition (MET) pathway. We then progress to elucidating ribosome-induced cancer cell multipotency, the associated stress, and the EMT pathway. In the final section of this review, we hypothesize a possible mechanism for ribosome-mediated cell fate reversal in light of our current findings.

## 2. Ribosome Incorporation for the Generation of Multipotent Cells from Somatic Cells

### 2.1. Ribosomes Are the Bacteria-Derived Factors Inducing Cluster Formation and Reprogramming

Endosymbiotic relationships led to the evolution of eukaryotic cells from prokaryotic organisms, and many eukaryotic organelles arose from the engulfment of previously free-living prokaryotes that were close to bacteria [28]. The symbiotic relationship between prokaryotes and eukaryotes coexisted with the evolution of simple eukaryotes into complex animals. We previously demonstrated that lactic acid bacteria (LAB) can reprogram somatic cells, proving that bacteria can influence eukaryotic cell fate [29,30]. When we infected human dermal fibroblast cells (HDFs) with LAB in vitro, the cells (HDFs) accumulated and formed sphere-like shapes. However, the capacity of bacteria to induce sphere formation was dependent on a mild trypsinization step, as trypsinization increases membrane permeability. After 14 days of culture, sphere cells expressed pluripotency markers. They were able to differentiate into three germ-layer-derived cells, both in vitro and in vivo. Unlike iPSCs, these sphere cells expressed a subset of pluripotent markers, including NANOG, SOX2, OCT3/4, and TDGF1, but not GDF3, FGF4, REX1, or ECAT15. Another feature of these cells is their inability to proliferate, which is linked to the expression of senescent markers p15, p16, and ARF. Electron microscopy analysis of the sphere cells revealed that LAB were localized in the cytoplasm, implying that LAB in the culture found a mechanism to enter cells and induce multipotency. In a subsequent study, we revealed that the multipotency was caused by LAB ribosomes [16].

**Figure 1:**
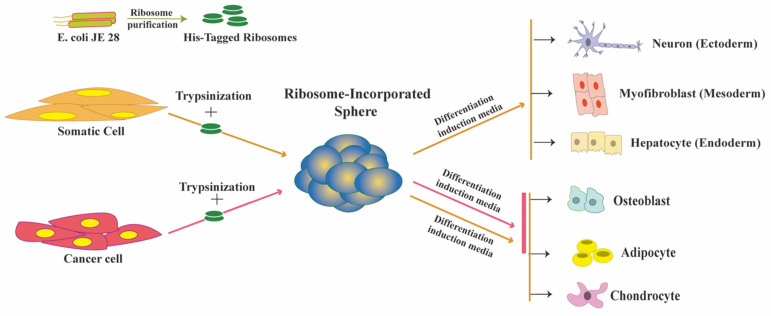
Ribosome incorporation leads to multipotency in somatic and cancer cells.

To identify the responsible factor, we fractionated the LAB cell lysate using column chromatography and evaluated each fraction for sphere-inducing ability on HDFs. Using mass spectrometry analysis, we found that the fraction with the highest activity contained ribosomal proteins. We used purified ribosomes from various sources (laboratory-generated and commercially available) and verified their ability to induce spheres, successfully establishing the ribosome as the responsible factor. In short, we introduced the pure ribosomes directly into the serum-free culture media containing trypsinized cells, and subsequently, the cells ingested these ribosomes by endocytosis. When we introduced different endocytosis blockers to the medium, sphere formation was inhibited, demonstrating that endocytosis is the ribosome uptake process. To assess the trypsinization effect, we introduced fluorescent beads to the trypsinized cell in a similar manner, which showed that trypsinization facilitates the up-take of these fluorescent beads by the cell. Therefore, trypsinization promotes ribosome uptake, which leads to sphere formation. Sphere formation is a well-established characteristic of stem cells [31,32]. We used ribosome-incorporated cell clusters (RICs) to name ribosome-induced spheres. RICs formed within one to two days of ribosome incorporation in HDFs. Exogenous ribosomes were found in the cytoplasm and nucleus of RICs, as detected by immune fluorescence analysis using antibodies specific to exogenous ribosome. We used tetra-(His)6-tagged ribosomes from E. coli JE28 bacteria (a generous gift from Dr. Ederth) for this purpose, which allowed us to trace the localization of exogenous ribosomes in HDFs. Genetically modified E. coli JE28 bacteria produce functioning ribosomes with a His-tag in the L12 ribosomal protein [33]. A subset of the RICs cells expressed pluripotency markers NANOG, POU5F1 (OCT4), SOX2, and SSEA4. NANOG and OCT4 expression levels in RICs were lower than those in iPSCs. We measured the global gene expression of RICs from day 0 to day 14, and the results indicated that the gene expression profile of RICs differed from that of control HDFs, iPSCs, and ES cells. In the differentiation media, the RICs differentiated into three germ-layer-derived cells. RICs also differentiated into adipocytes, osteoblasts, and chondrocytes. Figure 1 RICs, however, were unable to differentiate in vivo, unlike bacteria-induced spheres. The characteristics of RICs place them in an unusual category of multipotent cells. 

Surprisingly, ribosomes from various prokaryotic (Gram-positive and Gram-negative bacteria) and eukaryotic (yeast, mouse, and human) sources showed the sphere-forming effect on HDFs, suggesting a shared ribosomal element is involved in the mechanism. Although the composition and structure of ribosomes varies between prokaryotes and eukaryotes, thirty-four ribosomal proteins are conserved (15 small subunit proteins and 19 large subunit proteins) between them [34]. These proteins, however, are not fully conserved; therefore, the reprogramming function of the ribosome may be linked to sequence similarity.

Ribosome-mediated reprogramming does not seem to result from the translational activity of the incorporated exogenous ribosomes. The sphere-inducing potential of bacterial ribosomes was unaffected when gentamicin (a prokaryotic translation inhibitor) was added to the media [16,22].

Ribosomes require 15 mM magnesium to maintain their entire structure [35]. In the experiment for generating multipotent spheres, we excluded magnesium from the media, which would typically cause the exogenous ribosomal subunits to dissociate, supporting the idea that ribosomal protein(s) triggers the process. However, whether reprogramming activity requires the entire ribosomal proteins (RPs) or only a few of them remains unknown. In our research, exogenous ribosomes (independent of species variation) are responsible for the sphere formation effect. There is evidence that ribosomal proteins can function across species; rat ribosomal proteins P0, P1, P2, and RL12 can replace bacterial ribosomal proteins L10, L7, L12, and L11 that produce functioning hybrid ribosomes; and bacterial ribosome L11 can assemble with yeast ribosomes [36,37]. However, no information is currently available regarding the interaction of exogenous ribosomes inside the mammalian cells. Some reports have indicated that endogenous single-ribosomal proteins, in contrast, can carry out ‘extra-ribosomal functions’ away from the complex, modulating cellular homeostasis [20]. In the mammalian cell, RPS3 enters the nucleus and acts as a DNA endonuclease [38]. RPS3 can also bind the transcription factor NFκB in the nucleus and mediate specific interactions with the genome [39]. RPL10 acts as an antiviral component in the *Arabidopsis* plant; in humans, RPL10 also binds to and inhibits the function of JUN (a transcription factor subunit) [40,41]. RACK1, a non-permanent member of the ribosome, functions as a cell-signaling molecule [42,43]. Additionally, RPL13a controls the mRNA translation that is specifically responsible for the formation of the interferon-gamma-activated inhibitor of translation (GAIT) complex [40]. During ribosome-mediated reprogramming, exogenous His-tagged ribosomal proteins L12 have been found in the nucleus of the reprogrammed cell and might have a similar functional role interacting with transcription factors. The extra-ribosomal functions of some ribosomal proteins have also been implicated in the processes of development, immune response, and disease, suggesting ribosomal proteins’ ability to modulate different cellular processes [21]. In development, RPL38 shows Hox gene translation specificity that influences body patterning [44]; RPL12 takes part in T-cell development, and RPL3, RPL6, and RPL23A contribute to development of the pancreas [45,46,47]. RPL13A is a regulator of the interferon-gamma-mediated inflammatory response [40]. RPS9 promotes CDK1 expression, and RPL19 promotes cyclin D1 expression in cancer cells [48,49]. RP(L5, L6, L11, L23, L26, L37, S3, S7, S14, S15, S20, S25, S26, S27, S27A, S27L) is linked to the activation of tumor suppressor P53 [21]. However, it is unclear whether these ribosomal proteins function as free ribosomal proteins separately from their respective complexes.

### 2.2. Downstream Events Underlying Cell Reprogramming Triggered by Ribosomes

Molecular analysis revealed that RICs undergo partial mesenchymal to epithelial transition (MET) during the acquisition of multipotency and trigger cell stress. MET is essential in the process of iPSC generation and occurs at the early stage of reprogramming, where cellular stress is typically a hindrance [50,51]. However, it appears that cell stress and multipotency co-exist in RICs. Transcriptome analysis has highlighted the upregulation of several genes associated with cellular stress pathways, listed in Table 1.

#### 2.2.1. RICs Multipotency Is Coupled with Cellular Stress

**Figure 2:**
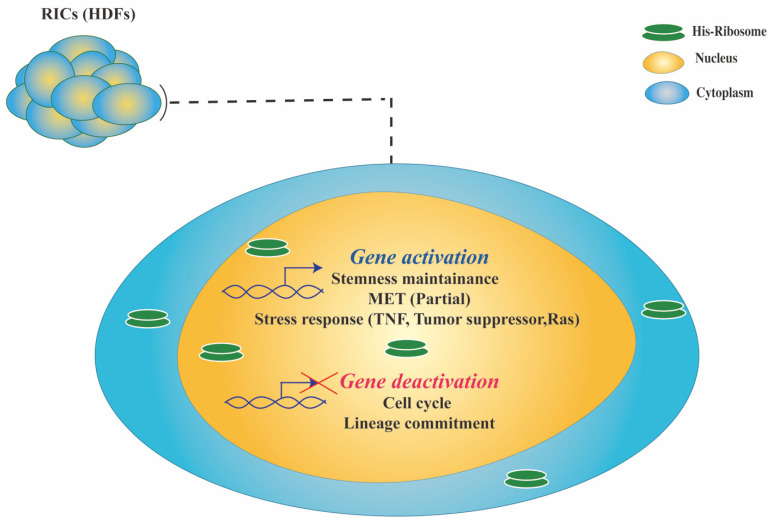
Summary of ribosome incorporation-mediated gene regulation in RICs.

Tumor suppressors (TSs) and tumor necrosis factors (TNFs) have well-established functions in cell-cycle and senescence regulation [60,66]. Many TSs are involved in the NF-kappaB pathway, a primary response to cell stress that maintains cell survival [67]. Another family of stress-associated proteins are Ras proteins, which are members of the small GTPase family and function as a molecular switch in cellular signaling and regulate genes involved in cell proliferation, differentiation, and survival [68]. Cell cycle analysis shows RICs possess cells with different cell-cycle phases (G0/G1, S/G2, and M) in a mosaic pattern, with a high number of S/G2 phase cells and a low number of M phase cells. RICs also exhibit peripheral CDC27 expression and a low number of apoptotic cells (ssDNA, DNA damage marker), suggesting cell-cycle progression is not arrested for some cells. RNA-seq analysis of RICs indicated that many cell-cycle-specific genes were downregulated. Furthermore, RICs exhibited significant upregulation of a subset of (TS) and (TNF) genes that may be involved in the stress response and cellular senescence-like state (Figure 2). Several Ras-related genes were also upregulated in RICs, though some were also downregulated. It is difficult to establish a direct link between exogenous ribosomes and these upregulated genes (TS, TNF, Ras) in RICs because the molecular mechanism is unknown. In addition, although the role of these upregulated TS, TNF, and Ras genes in cancer cells is well-understood, little is known regarding their function in HDFs. However, based on their known functions in regulating the cell cycle and senescence, their role in the RICs senescence-like state can be inferred. It is worth noting that RICs retain their multipotency during the senescence-like state, and the state is reversed in the differentiation induction medium, resulting in proliferative differentiated somatic cells.

#### 2.2.2. RICs Multipotency Induction Involves a Partial MET Process

MET is the reverse process of epithelial-to-mesenchymal transition (EMT). MET is critical for inducing cell reprogramming [69]. Indicators of MET include the upregulation of E-cadherin and the downregulation of N-cadherin, Vimentin, Twist1, and Snail1 [70]. The formation of RICs has also been identified to influence some of these markers; cell motility and mesenchymal cell morphology were lost during the formation of RICs. RICs did not express Snail1 and had a lower Vimentin and CDH (N-Cadherin) expression than normal HDFs. Twist1 was, however, expressed in RICs. These findings suggest the presence of an alternative pathway and the occurrence of a partial MET during the formation of RICs.

## 3. Ribosome Incorporation Modulates Cancer Cell Fate

The incorporation of ribosomes in cancer cells elicits a response similar to the incorporation of ribosomes in somatic cells. We recently showed that ribosome incorporation causes sphere formation, multipotency, and cell-cycle arrest in non-small-cell lung cancer A549 and gastric tubular adenocarcinoma cells H-111-TC [23]. We also found that ribosome incorporation causes partial EMT (epithelial–mesenchymal transition) and cell-cycle arrest in the MCF7 breast cancer cell line [24]. These studies lend credence to the possibility of a stress-associated mechanism of exogenous ribosomes. We named spheres generated from cancer cells as ribosome-incorporated cancer cell clusters (cRICs). cRICs from A549 and H-111-TC transdifferentiated into adipocyte and osteoblast in the differentiation induction medium (Figure 1).

### 3.1. Ribosomes Induce Cellular Stress in Cancer Cells

Cyclin D1 is a crucial marker for cell-cycle progression, and the reduction of cyclin D1 levels leads to cell-cycle arrest [71]. P53 (a tumor suppressor protein) stimulates P21, which inhibits cyclin D1 and stops the cell cycle [72]. In addition to P21, P53 can also inhibit cell-cycle progression through P53-targeted genes. P21-deficient cells show the slight activity of P53-mediated G1 cell-cycle arrest [72,73]. cRICs, such as RICs, do not proliferate. The majority of the cRICs cells are in the senescent G0 phase and re-enter the cell-cycle during differentiation. In the MCF7 cRICs, the P53 expression increased, but the P21 expression was unrelated to P53. However, cyclin D1 levels in MCF7 and A549 cRICs decreased after ribosome incorporation, suggesting an alternative P53-mediated pathway present in cRICs. Cellular stress can induce autophagy by upregulating NF-kappaB and LC3 a/b [74]. Ribosome-incorporated MCF7 cells trigger the autophagy upregulating the NF-kappaB precursor, activated NF-kappaB, and LC3 a/b [24]. These molecular stresses lead to cell-cycle arrest in the cancer cell, which may have an implication for cancer therapy. Transplanting A549 cRICs into a mouse model caused tumor formation, suggesting that in vivo condition (with its complex growth factors) can reactivates these cells. When we injected ribosomes directly into the tumor, we found no difference in tumor volume compared with the control group, probably because the ribosome was diluted and was not sufficiently taken up by the tumor cell. In the future, we will conduct vehicle-based delivery of free ribosomes into tumor cells, which will hopefully show in vivo cell-cycle arrest and reprogramming.

### 3.2. Ribosomes Induce Partial EMT in Cancer Cells

**Figure 3:**
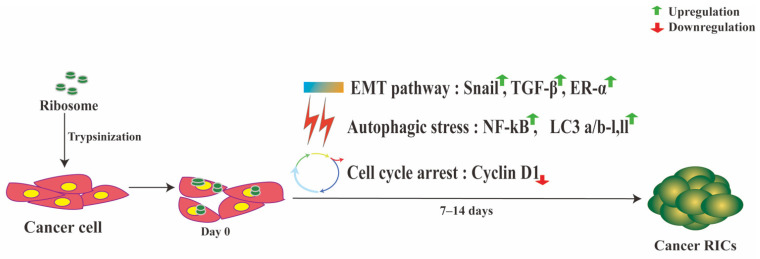
Ribosomes stop cancer cell proliferation through stress.

MCF7 cRICs showed upregulation of TGF-b1 and Snail during sphere formation, which is compatible with EMT [24]. TGF-b, Snail, and E-cadherin are well-known EMT inducers, where Snail and TGF-b downregulate E-cadherin during the EMT process [75,76]. Snail activates the PI_3_ kinase/Akt pathway in the TGF-b-induced EMT process [77,78]. However, E-cadherin expression in MCF7 cRICs did not correlate with TGF-b or Snail expression and remained elevated during sphere formation, implying that partial EMT occurs in cRICs. EMT is a critical process in embryogenesis, cancer metastasis, and reprogramming [77]. Thus, EMT might aid the reprogramming process in cRICs as well (Figure 3). Compare to RICs, the cRICs showed an opposite EMT/MET transition, suggesting that the response of ribosome incorporation to reach multipotency is dependent on cell type. However, exactly what property determines such a difference is unknown. A detailed temporal analysis of the EMT/MET markers for both cell types may provide an answer in the future.

## 4. Mechanistic Insights in Ribosome-Induced Multipotency

**Figure 4:**
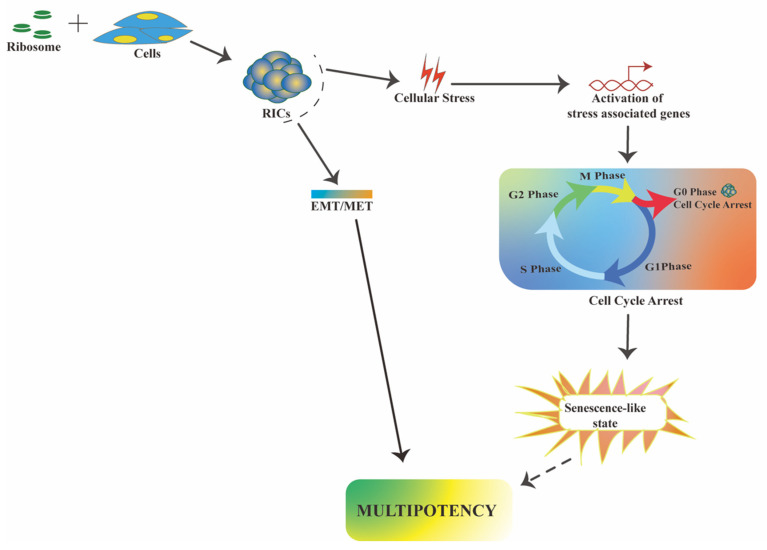
Proposed pathway for ribosome-mediated cellular multipotency.

Although normal somatic cells and cancer cells have very different molecular characteristics, ribosome incorporation appears to affect them in the same way. Exogenous ribosomes are found in the nucleus and cytoplasm of both types of cells. Persistent nuclear localization indicates that the exogenous ribosomal proteins can pass through the nuclear pore and may have a nuclear function. Therefore, similar to extra-ribosomal functions of the single ribosomal protein mentioned above, exogenous ribosomes may interact with the transcription factors. Thus, it is theorized that such an interaction somehow allows ectopic expression of stemness-specific transcription factors, such as Oct4, Nanog, and Sox2. The cell proliferation rate and internal ribosome levels may impact the efficiency of multipotency induction by the incorporated ribosome. Cancer cells have a high rate of ribosome biogenesis due to their constant need for proteins and rapid proliferation [79,80]. In comparison to normal HDFs, cancer cells require a large number of ribosomes and a long time period for sphere formation. Ribosome incorporation shows a common molecular pattern during multipotency induction in cancer and somatic cells. Both cells undergo cell-cycle arrest and elicit a stress response. It opens up the possibility of an exogenous ribosomal mechanism, triggering cellular multipotency via stress. Cellular stress can reprogram transcription and reorganize chromatin [81]. Therefore, ribosome-induced stress may facilitate specific expression of stemness genes that were previously silent. Several studies claim that cellular senescence is a barrier to efficient in vitro cell reprogramming [51]. Nevertheless, the relationship between cellular senescence and reprogramming is not always straightforward. A study in mice found that cellular senescence provides essential signaling for in vivo cell reprogramming and that Yamanaka factors induce both senescence and pluripotency [82]. Ribosome-induced RICs have an heterogeneous expression of stemness and cell-cycle phases. It is possible that fully senescent cells in the RICs provide a similar signal, which allows the reprogramming of other cells. However, extensive research is necessary to prove this hypothesis. Ribosome incorporation also affects EMT/MET pathway-related markers in both cells; however, the process is reversed based on cell character. Moreover, none of the pathways are fully activated by the exogenous ribosome, indicating the presence of an alternate mechanism. Based on these findings, we propose that ribosome-induced multipotency is aided by cellular stress and is preceded by a partial EMT/MET process (Figure 4).

## 5. Conclusions

Ribosome-mediated multipotency introduced a new paradigm in cell reprogramming study. It establishes a method for producing multipotent cells (RICs) capable of differentiating into derivates of all three germ layers. A senescence-like state and cell stress are typical responses to ribosome incorporation during multipotency induction, suggesting a stress-associated mechanism for the exogenous ribosomes; consequently, extensive further study is necessary. Senescence and multipotency induced by exogenous ribosomes have the potential to be used in regenerative medicine and cancer therapy. However, the inability of self-renewal limits RICs from becoming stem cells. RICs are more of a proxy state for quiescent stem cells. It is difficult to speculate on the mechanism of exogenous ribosomes at this moment because no other study is available. Future research into the exogenous ribosome interactome, temporal gene expression, and epigenetic changes will help us understand the underlying mechanism.

## Figures and Tables

**Table 1:** Stress-associated genes found to be upregulated in RICs by RNA-seq analysis. Gene expression was compared between day 0 and day 14 after ribosome incorporation [16]. Upregulation cut off value = 1.2 (Fold change).

Category	Gene	Gene Upregulation in RICs (Fold Change)	Stress-Related Functions
Tumor suppressor/growth suppressor	TP53I11, tumor protein p53 inducible protein 11	2.58	Metastasis and EMT inhibition [52], Apoptosis [53]
	CDKN2B, cyclin-dependent kinase inhibitor 2B (p15)	1.27	Inhibits CDK4, growth suppression [54]
	TP63, tumor protein 63	3.0	Growth suppression, senescence, survival [55]
	MTUS1, microtubule-associated tumor suppressor	1.47	Growth suppression and senescence [56]
	TUSC1, tumor suppressor candidate 1	1.47	Growth suppression [57]
Tumor necrosis factor	C1QTNF4, C1q, and tumor necrosis factor-related protein 4	3.04	Activates the NF-kappaB pathway, survival [58]
	TNFAIP3, tumor necrosis factor, alpha-induced protein 3	1.68	Regulates the NF-kappaB pathway, apoptosis [59]
	TNFAIP6, tumor necrosis factor, alpha-induced protein 6	2.59	Activates the NF-kappaB pathway [60]
	TNFSF10, tumor necrosis factor (ligand) superfamily, member 10	4.04	Apoptosis [61]
Ras-associated genes	RASD1, Ras dexamethasone-induced 1	2.47	Stress response, suppression of aberrant cell growth, proliferation [62]
	RRAGD, Ras-related GTP binding D	2.57	Stress response, regulation of mTORC1 signaling [63]
	RRAD, Ras-related associated with diabetes	1.47	Regulation of cell cycle, apoptosis [64]
	RHOJ, Ras homolog gene family, member J	3.98	Proliferation [65]

## Data Availability

Not applicable.
